# Association of vitamin E intake in diet and supplements with risk of dementia: A meta-analysis

**DOI:** 10.3389/fnagi.2022.955878

**Published:** 2022-08-01

**Authors:** Rangyin Zhao, Xiaoyong Han, Hongxia Zhang, Jia Liu, Min Zhang, Weijing Zhao, Shangrong Jiang, Ruilin Li, Hui Cai, Hong You

**Affiliations:** ^1^Gansu University of Chinese Medicine, First Clinical Medical College, Lanzhou, China; ^2^Sino-French Department of Neurological Rehabilitation, Gansu Provincial Hospital, Lanzhou, China; ^3^Graduate School, Ning Xia Medical University, Yinchuan, China; ^4^Lanzhou No.1 People's Hospital, Lanzhou, China; ^5^General Surgery Clinical Medical Center, Gansu Provincial Hospital, Lanzhou, China; ^6^Key Laboratory of Molecular Diagnostics and Precision Medicine for Surgical Oncology in Gansu Province, Gansu Provincial Hospital, Lanzhou, China; ^7^First Clinical College of Medicine, Lanzhou University, Lanzhou, China; ^8^NHC Key Laboratory of Diagnosis and Therapy of Gastrointestinal Tumor, Gansu Provincial Hospital, Lanzhou, China

**Keywords:** vitamin E, diet, supplements, dementia, risk, meta-analysis

## Abstract

**Background:**

Dementia is a chronic progressive neurodegenerative disease that can lead to disability and death in humans, but there is still no effective prevention and treatment. Due to the neuroprotective effects of vitamin E, a large number of researchers have explored whether vitamin E can reduce the risk of dementia. Some researchers believe that vitamin E can reduce the risk of dementia, while others hold the opposite conclusion. We therefore performed a meta-analysis to clarify the relationship between them.

**Methods:**

We searched PubMed, Embase, and Web of Science databases for articles on the connection of dietary and supplementation vitamin E with dementia risk from inception through April 2022 using the main keywords “dementia,” “Alzheimer's disease,” “vitamin E,” and “tocopherol,” and used a random-utility model for pooled effect sizes. Odds ratios (OR) and 95% confidence intervals were derived using lower and higher doses as contrasts. Obtained data were shown and assessed using Stata12.0 free software.

**Results:**

We included 15 articles in sum. Among them, there were nine articles containing AD. By comparing the highest intake with the lowest intake, Combined ORs for high intake were as follows: dementia (OR = 0.79, 95% CI 0.70–0.88 *I*^2^ = 35.0%), Alzheimer's disease (OR = 0.78, 95% CI 0.64–0.94 *I*^2^ = 36.9%). Subgroup analyses were also performed by study type, diet and supplementation, and NOS score.

**Conclusions:**

High vitamin E intake from diet and supplements significantly reduces the risk of dementia and Alzheimer's disease.

## Introduction

Dementia is a condition that causes gradual cognitive decline (Chertkow et al., 2013). With the aging of the population and the growth of the population, the prevalence of dementia is expected to increase, and it is estimated that the number of people with dementia worldwide will increase from 570,000 in 2019 to 1.58 million in 2050 (GBD 2019 Dementia Forecationg Collaborators, 2022). Dementia has emerged as a leading cause of disability, loss of independent living, and death in older adults, and places a heavy burden on patients, families, and society (Livingston et al., 2020; Malik et al., 2021), while there is still no clear cure for dementia in terms of treatment (Winblad et al., 2016). Dementia includes Alzheimer's disease (AD), vascular dementia (VD), and other forms of dementia, with AD being the most common, followed by VD (Zhang et al., 2021). Dementia is caused by both genetic and environmental factors. Age, smoking, diabetes, obesity, and hypertension are all variable risk factors for dementia (Sindi et al., 2018), and diet has also been shown to have some effect on the development of dementia (Morris, 2016). Therefore, we can prevent the occurrence of dementia by changing the diet and lifestyle.

Fruits and vegetables are indispensable foods in life and provide the body with a large number of nutrients. Among them, vitamins are more common, and vitamin E has also been gradually found to prevent various diseases (Sharma et al., 2021). Some studies have found that diet is considered one of the key factors in reducing the risk of dementia (Sindi et al., 2018; Licher et al., 2019). Vitamin is an organic substance that maintains the normal life activities of the human body and supports basic cellular functions and plays an essential role in various basic metabolic processes in the body (Tardy et al., 2020). However, vitamin E is less abundant in the body and is mostly consumed through the diet (Del Mondo et al., 2020). Vitamin E is widely present in various foods and belongs to fat-soluble vitamins, and its main active form in tissues is α-tocopherol (αT; Farina et al., 2017). Vitamin E not only has a strong antioxidant capacity, but also has the properties of reducing cholesterol and neuroprotection, thus exerting a protective effect on the brain (Jiang, 2014; Browne et al., 2019).

The association between diet and the risk of dementia has been extensively explored over the last few decades, with people who consume large amounts of vegetables and fruits having a markedly lower risk of developing dementia (Dai et al., 2006; Barberger-Gateau et al., 2007; Hughes et al., 2010). Vegetables and fruits contain a large number of vitamins, minerals, cellulose, etc., which play an important role in human health (Wiseman, 2008). Vitamin E is an antioxidant that can reduce the risk of dementia onset (Giraldo et al., 2014; Eshkoor et al., 2015; Farina et al., 2017; Lakhan et al., 2021). However, other studies have demonstrated that vitamin E intake does not lower the risk of dementia (Kryscio et al., 2017). Therefore, we determine whether dietary vitamin E or supplements can reduce the risk of dementia and provide more reliable evidence for the prevention of dementia by timely integrating the latest views and related evidence.

## Materials and methods

### Search strategy for literature

This meta-analysis was conducted and reported according to the Preferred Reporting Items for Systematic Reviews and Meta-Analyses (PRISMA) guidelines. Two authors (Rangyin Zhao and Xiaoyong Han) conducted a paper search for the association of vitamin E and active ingredients with dementia risk using EMBASE, Web of Science and PubMed databases, respectively. The database was searched using keywords/titles/abstracts searching for the keywords: “vitamin E” or “alpha-tocopherol” or “α-tocopherol” or “nutrients” combined with “dementia” or “amentia” or “amentias” or “Alzheimer's Disease” or “Alzheimer Dementia” or “Alzheimer Dementias” or “Dementia, Alzheimer.” The search was conducted from inception to April 2022 for all relevant articles. In addition, we searched conference proceedings, meta-analyses, and references from other articles to avoid possibly missing research. We only included English literatures. Search disagreements between the two authors and a third author discuss decisions together.

### Inclusion and exclusion criteria

We developed the inclusion criteria for our study using PICOS principles. P (Participants): middle-aged and elderly population without dementia and cognitive impairment; I (intervention): high intake of vitamin E from diet or supplements; C (control): low intake of vitamin E from diet or supplements; O (outcome): risk of dementia. S (study design): case-control study or cohort study. Studies that met the following criteria were included: (1) patients with a definite diagnosis of dementia; (2) the type of study was a cohort or case-control study; (3) the association between diet or supplement vitamin E or tocopherol and the risk of dementia was studied and compared between high and low dose; (4) the study contained the following relevant data: relative risk (RR) or odds ratio (or) and 95% confidence interval; (5) the study subjects were humans, excluding experiments such as cells and animals. The exclusion criteria used were as follows: (1) no valid data could be extracted from the text; (2) duplicate study populations; (3) small sample size; (4) reviews, conferences, meta-analyses, letters, etc.

### Data extraction and quality evaluation

Two researchers independently performed quality assessment and extraction of relevant data for all included studies. Basic information for inclusion included: first author name, date of publication, country, sex, age, type of dementia, type of study, Sample Size of Study, RR or OR and 95% CI adjustment for covariates in multivariate analysis. Discrepancies between the two authors were resolved by discussion with the other authors. The Newcastle-Ottawa Scale (0–9) was used to evaluate the quality of the literature used.

### Statistical analysis

Effect sizes for all studies were RR or OR and 95% confidence intervals to analyze the connection of high-dose vitamin E or tocopherol intake with the risk of dementia. The effect sizes for assessing dementia risk were RR, OR for the cohort study and case-control study, respectively. Since the difference between the two effect sizes is small, the OR value is uniformly used to represent it for the convenience of combining and calculating the study results. We used a random-effects model to count effect sizes. The results of meta-analysis were presented as forest plots, and publication bias was assessed by Begg's test and funnel plots. In addition, sensitivity analysis assessed the stability of the meta-analysis by eliminating each study one by one. All statistical analyses were calculated using Stata12.0, with *p* < 0.05 indicating a statistically significant difference.

## Results

### Screening process for eligible literatures

**Figure 1:**
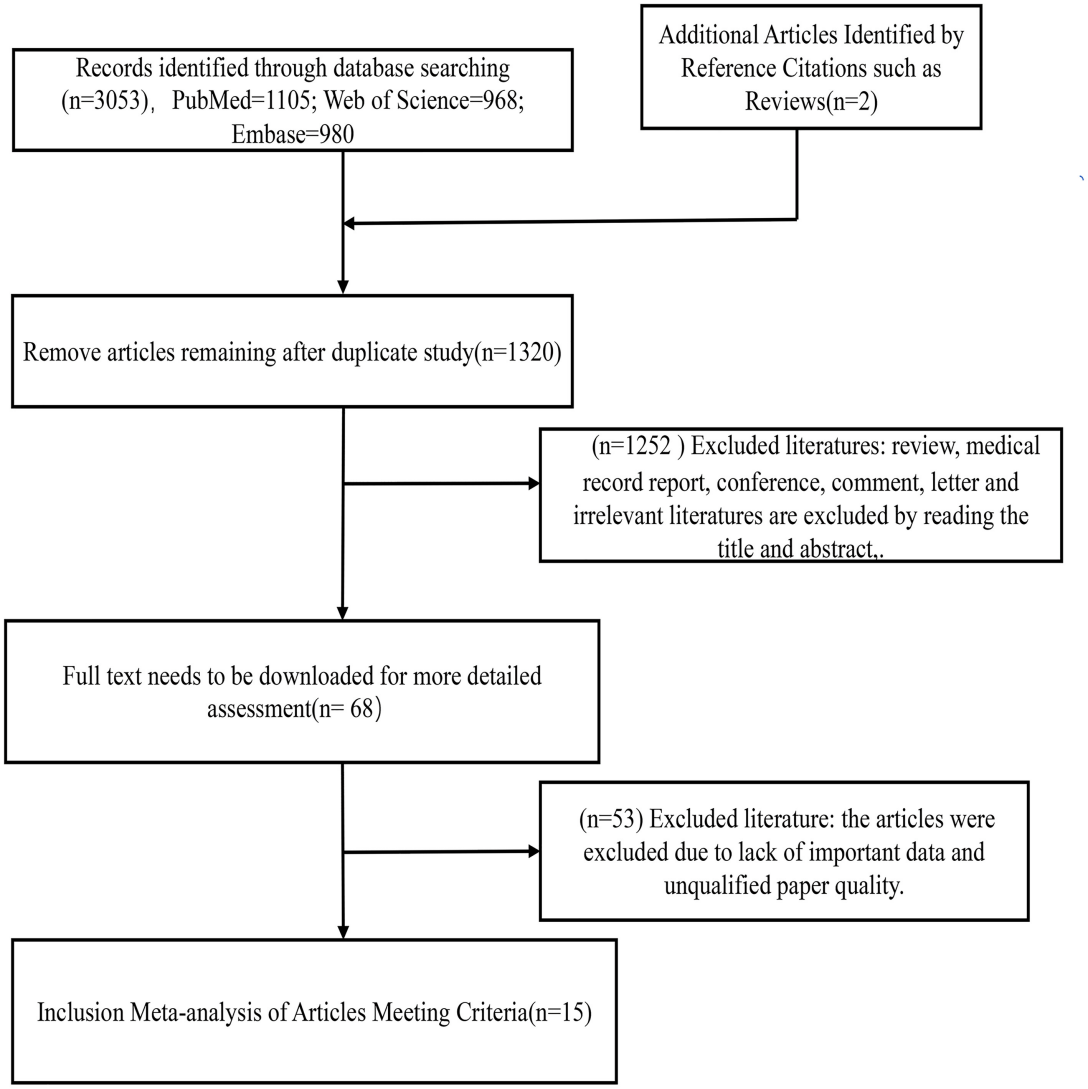
Flow diagram of this meta-analysis.

Three thousand and fifty-three literatures were retrieved through three English database retrieval systems, which were PubMed (*n* = 1,105), Embase (*n* = 980), and Web of Science (*n* = 968). Two useful original articles were obtained by searching the citations of articles such as reviews or meta-analyses. After removing duplicates, 1,320 articles remained. Reviews, case reports, conferences, comments, letters, and irrelevant articles were excluded by reading the titles or abstracts, for a total of 1,252. The additional 68 essays have been assessed by downloading the full text and articles with missing raw data were excluded. The remaining 15 studies that met the inclusion criteria and NOS score were included in the meta-analysis. The above retrieval process is shown in Figure 1.

### Characteristics of included research

The features of the 15 papers included in the meta-analysis are shown in Table 1. We included 15 articles in overall, eight on diet, six on supplements, and one on a combination of diet and supplements. These included 13 cohort studies with 46,968 participants and 6,046 dementia patients after 4–23 years of follow-up; two case-control studies with 3,157 controls and 3,459 patients. We extracted studies on AD with a total of nine articles. Five of them were about diet, three were about supplements, and one was related to dietary combined supplements. Eight cohort studies with 28,530 participants followed for 4–23 years yielded 2,852 dementia patients; one case-control study with 2,999 controls and 3,385 patients. The studies included in this meta-analysis were published between 1983 and 2022. In addition, 11 studies were conducted in North America, 3 in Europe, and 1 in Asia. The main nutritional species investigated were vitamin E or tocopherol. Included with the articles were corrected for multivariate variables such as age, education, sex, smoking status, alcohol intake, body mass index, and a history of cardiovascular and cerebrovascular illness. The literary NOS quality score ranged from 6 to 8. Study characteristics for all included Mate analyses are presented in Table 1.

**Table 1:** Characteristics of included studies.

**References, Country**	**Mean age,** **Sex**	**Subtypes of dementia**	**Type of study**	**Sample size**	**Diet/Serum/** **Supplements**	**Dose: highest comparison lowest**	**OR (95%Cl)**	**Adjustment for covariates**	**NOS score**
Aoki et al. (2021), Japanese	40–64, /	Dementia	Cohort study	3,739/670	Diet	Four- vs. One-fold	0.50 (0.34–0.74)	Age, sex, smoking, energy intake, region, history of stroke, docosahexaenoic acid, and docosahexaenoic acid	7
Gray et al. (2008), USA	≥65, /	Dementia	Cohort study	964/122	Supplements	/	0.98 (0.77–1.25)	age, sex, education, exercise, smoking status, self-reported health, and coronary heart disease	8
Engelhart et al. (2002), Netherlands	≥55, /	Dementia	Cohort study	5,395/146	Diet	>15.5 vs. <10.5 mg/d	0.82 (0.66–1.00)	Age, sex, baseline Mini-Mental State Examination score, alcohol intake, education, smoking habits, number of pack-years smoked, body mass index, total energy intake, presence of carotid plaques, and use of antioxidant supplements	8
Olson (2000), USA	71–93, Male	Vascular dementia/AD/Other dementia	Case-control study	3,385/2,999	Supplements	/	0.58 (0.17–2.01)/ 1.03 (0.47–2.25)/ 0.60 (0.23–1.59)	Age, years of formal education, history of stroke, and apoE phenotype	8
Basambombo et al. (2017), Canada	>65, /	AD/Other dementia	Cohort study	5,269/ 821	Supplements	/	0.62 (0.39–0.98)/0.61 (0.41–0.90)	Age, gender and education, ever regular smoking, alcohol drinking, regular physical activity, NSAID use, history of diabetes, and vascular risk factors	7
Luchsinger et al. (2003), USA	≥65, /	AD	Cohort study	4,023/242	Diet	Four- vs. one-fold	0.98 (0.67–1.44)	Age, sex, APOE 4 allele presence, smoking status, and years of education	6
Morris et al. (2002), USA	≥65, /	AD	Cohort study	815/131	Diet	10.4–43.0 vs. <7.0 IU/d	0.30 (0.10–0.92)	Age, sex, education, APOE 4 status, race, an interaction term between race and APOE 4, and period of observation	8
Zandi et al. (2004), USA	≥65, /	AD	Cohort study	5,092/355	Supplements	/	0.53 (0.20–1.12)	Age, the squared deviation of age from the population median, sex, education and APOE 4	6
Devore et al. (2010), Netherlands	>55, /	Dementia/AD	Cohort study	4,751/407	Diet	18.5 vs.9.0 mg/day	0.75 (0.58–0.97)/0.75 (0.57–1.00)	Age, education, APOE ε4 genotype, total energy intake, alcohol intake, smoking habits, and BMI	6
Corrada et al. (2005), USA	/, /	AD	Cohort study	579/57	Diet+ Supplements	Three- vs. one-fold	0.62 (0.32-1.20)	Gender, education, and baseline age and caloric intake	8
Morris et al. (2005), USA	71–93, Male	AD	Cohort study	1,041/162	Diet	The difference between high and low intake was 5 mg/d	0.74 (0.62-0.88)	Age, sex, race, education, APOE 4, the interaction between race and APOE, frequency of participation in cognitive activities, time from determination of disease-free status to clinical evaluation of incident disease, and intakes of saturated fat, trans unsaturated fat (g/d), and docosahexaenoic acid	7
Laurin et al. (2004), USA	45–68, Male	Dementia /AD/Vascular dementia	Cohort study	2,459/235	Diet	29.9 vs. 3.8 mg/d	1.33 (0.90–1.96)/1.58 (0.87–2.85)/1.07 (0.41-2.78)	Age, education, smoking status, alcohol intake, body mass index, physical activity, systolic and diastolic blood pressures, year of birth, total energy intake, cholesterol concentration, history of cardiovascular disease, supplemental vitamin intake, and apolipoprotein E e4	7
Kryscio et al. (2017), USA	≥62, /	Dementia	Cohort study	1,799/71	Supplements	400 IU/d vs. no use	0.88 (0.64–1.20)	Age, Black ethnicity, APOE ε4 carrier status, college education, baseline MIS score	8
von Arnim et al. (2012), Germany	65–90, /	Dementia	Case-control study	74/158	Diet	0.51 vs. 0.08 μM	0.71 (0.24–2.13)	School education, BMI, alcohol consumption, smoking status, and current dietary supplement use	6
Paganini-Hill et al. (2016), USA	≥90, /	Dementia	Cohort study	587/293	Supplements	/	0.86 (0.67–1.10)	Age, sex and education	6

### Dietary or supplemental vitamin E in relation to risk of dementia

**Figure 2:**
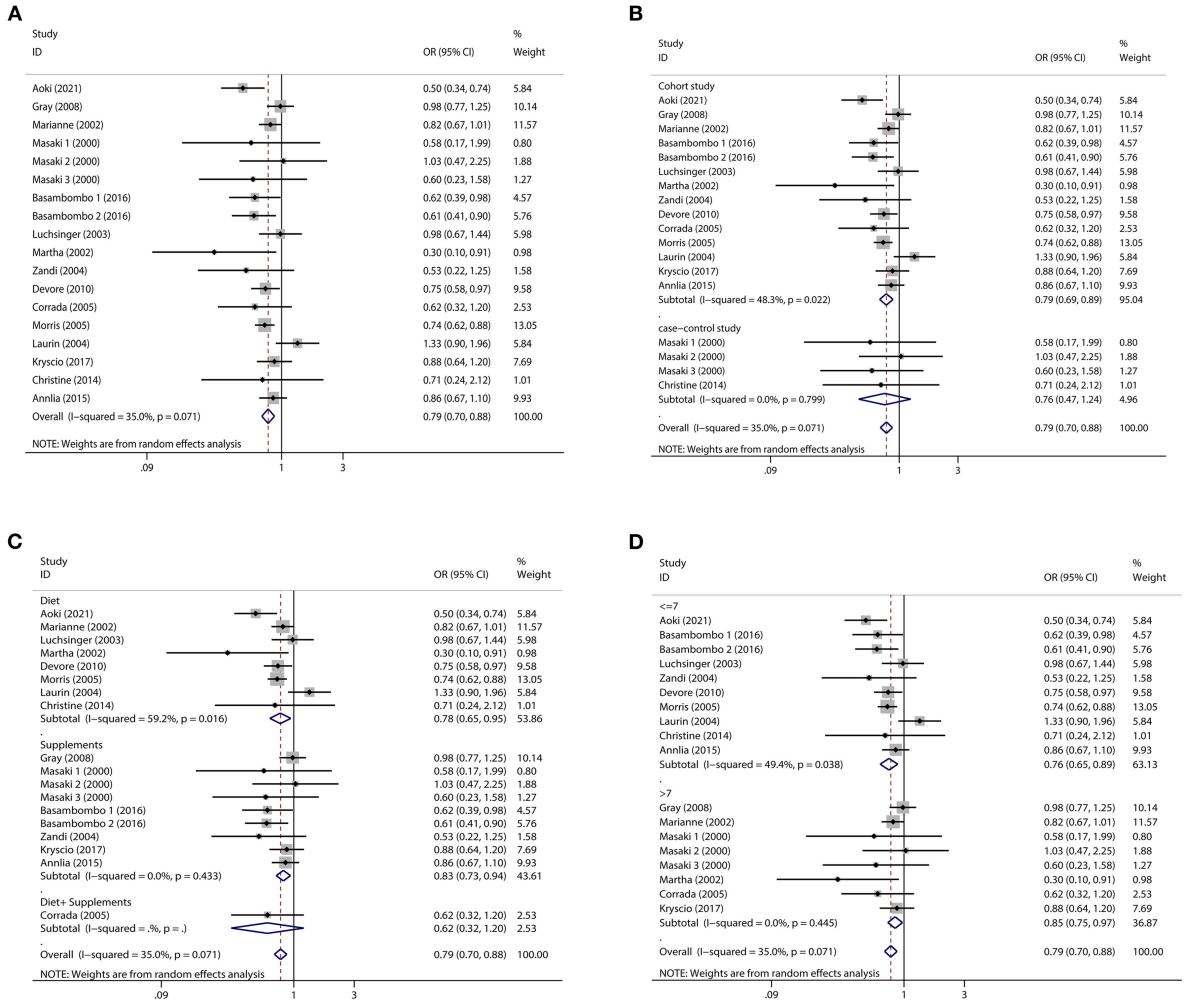
Forest plots and subgroup analysis plots of high intake of dietary or supplemental vitamin E and risk of dementia. **(A)** Forest plot. **(B)** Subgroup analysis by study type. **(C)** Subgroup analysis by diet and supplements. **(D)** Subgroup analysis by NOS quality score.

**Figure 3:**
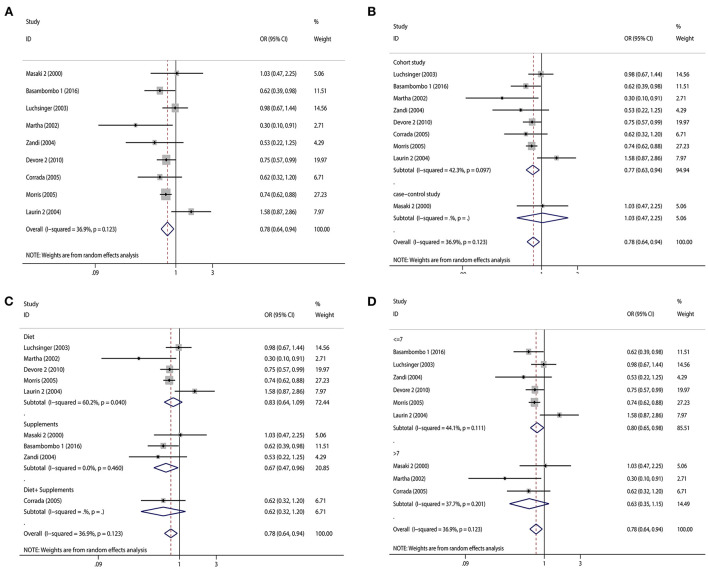
Forest plots and subgroup analysis plots of high intake of dietary or supplemental vitamin E and risk of AD. **(A)** Forest plot. **(B)** Subgroup analysis by study type. **(C)** Subgroup analysis by diet and supplements. **(D)** Subgroup analysis by NOS quality score.

Regarding diet and supplements, there were 15 research containing 18 pieces of data that were included in our study. A high intake of diet or vitamin E supplements decreased the risk of dementia by 21% (OR = 0.79, 95% CI 0.70–0.88, Figure 2A), with a statistically significant difference (*p*_*t*_ = 0.000). The heterogeneity was low (*I*^2^ = 35.0%, *p* = 0.071). When subgroup analyzed by study type, cohort studies (OR = 0.79, 95% CI 0.69–0.89, Figure 2B) showed that high vitamin E intake dramatically decreased the risk of dementia. Case-control studies (OR = 0.76, 95% CI 0.47–1.24, Figure 2B) tended to reduce the risk of dementia, but this was not statistically significant. We also performed subgroup analyses according to diet and supplement, and we found that both diet (OR = 0.78, 95% CI 0.65–0.95, Figure 2C) and supplement (OR = 0.83, 95% CI 0.73–0.94, Figure 2C) high vitamin E intake reduced the risk of dementia. When we did subgroup analysis according to NOS score, studies with scores >7 (OR = 0.85, 95% CI 0.75–0.97, Figure 2D) and ≤ 7 (OR = 0.76, 95% CI 0.65–0.89, Figure 2D) significantly reduced the risk of dementia. We screened some studies on AD according to dementia classification and we found a significant inverse association between vitamin E intake and AD risk (OR = 0.78, 95% CI 0.64–0.94, Figure 3A), with a statistically significant difference (*p*_*t*_ = 0.000). The heterogeneity was low (*I*^2^ = 36.9%, *p* = 0.123). Cohort studies (OR = 0.77, 95% CI 0.63–0.94, Figure 3B) showed a significant reduction in AD risk when subgroup analyses were performed according to study type. When subgroup analyses were performed by diet and supplement, both diet (OR = 0.83, 95% CI 0.64–1.09, Figure 3C) and supplement (OR = 0.67, 95% CI 0.47–0.96, Figure 3C) reduced the risk of AD and diet was not statistically significant. When performing subgroup analysis by NOS score, studies >7 (OR = 0.63, 95% CI 0.35–1.15, Figure 3D) and ≤ 7 (OR = 0.80, 95% CI 0.65–0.98, Figure 3D) both significantly reduced the risk of AD, but studies >7 were not statistically significant. Results of a meta-analysis of diet and supplements with risk of dementia are shown in Table 2.

**Table 2:** Meta-analysis of diet and supplements with risk of dementia.

							**Heterogeneity**	
**Dementia/AD**	**Diet and supplements vitamin E**	**Studies (** ***n*** **)**	**OR**	**95% CI**	***P*** **-value**	**Model**	***I*^2^** **(**%**)**	***P*** **-value**
	Vitamin E	15	0.79	0.70–0.88	0.012	Random	35.0	0.071
	Dietary Vitamin E	8	0.78	0.65–0.95	0.004	Random	59.2	0.016
	Vitamin E supplements	9	0.83	0.73–0.94	0.000	Random	30.6	0.173
Dementia	Cohort study	14	0.79	0.69–0.89	0.000	Random	48.3	0.022
	Case-control study	4	0.76	0.47–1.24	0.274	Random	0.0	0.799
	>7	8	0.85	0.75–0.97	0.017	Random	0.0	0.445
	< =7	10	0.76	0.65–0.89	0.000	Random	49.4	0.038
	Vitamin E	9	0.78	0.64–0.94	0.001	Random	36.9	0.123
	Dietary Vitamin E	5	0.83	0.64–1.09	0.182	Random	60.2	0.040
AD	Vitamin E supplements	3	0.67	0.47–0.96	0.031	Random	0.0	0.460
	Cohort study	8	0.77	0.63–0.94	0.010	Random	42.3	0.097
	>7	3	0.63	0.36–1.15	0.136	Random	37.7	0.201
	< =7	6	0.80	0.65–0.98	0.035	Random	44.1	0.111

### Publication bias and sensitivity analysis

**Figure 4:**
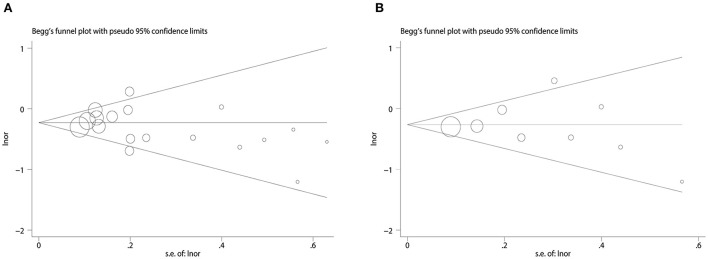
Publication bias begg's funnel plot. **(A)** Funnel plot for combined dietary and supplement outcomes with dementia risk. **(B)** Funnel plot for combined dietary and supplement outcomes with AD risk.

**Figure 5:**
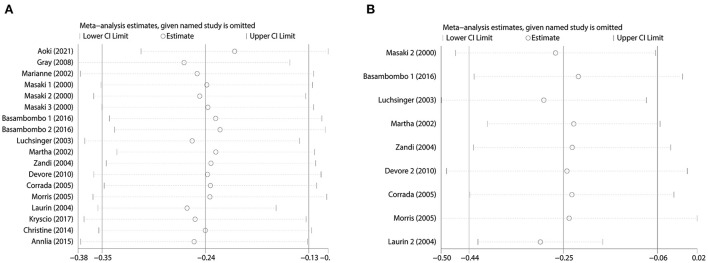
Sensitivity analysis. **(A)** Sensitivity analysis of combined dietary and supplement use and risk of dementia. **(B)** Sensitivity analysis of combined dietary and supplement use and risk of AD.

We used Begg's funnel plot and Begg's test to detect whether there was a significant publication bias. The results of Begg test for dementia (Figure 4A): (Pr > | z | = 0.405), Begg test for AD (Figure 4B): (Pr > | z | = 0.348), and Begg's funnel plots of dementia and AD were evenly distributed on both sides (Pr > | z | > 0.05), showed no Significant publication bias. In addition, we evaluated the stability of the meta-analysis by deleting studies one by one and re-combining ORs for sensitivity analysis (Figures 5A,B), and the pooled ORs fluctuated within a certain range after deleting each study, indicating that the results of this meta-analysis were stable. In summary, the conclusions of our study are relatively reliable.

## Discussion

Vitamin E is a fat-soluble antioxidant that plays an important role in human life activities (Lloret et al., 2019). Due to its antioxidant and cerebral protective effects, vitamin E can be used to prevent and treat neurodegenerative diseases (Brigelius-Flohé and Traber, 1999; Reiter et al., 2007; Jiang, 2014). Although some observational studies have reported that dietary intake or supplementation with vitamin E reduces the risk of dementia, there is also controversy between them, so we conducted a meta-analysis to comprehensively assess vitamin E and the risk of dementia. We included 15 clinical studies on dietary intake or supplementation with vitamin E and risk of dementia. We performed subgroup analysis based on dementia type, study category, NOS score.

The vast majority of studies included in our meta-analysis were prospective cohort studies, which greatly reduced recall excursions and were more credible, and the results showed that high dietary intake or vitamin E supplementation significantly reduced the risk of dementia and AD. Subsequently, we performed a subgroup analysis of diet and supplements and found that diet and supplements reduced the risk of dementia, but diet was superior, whereas for AD, supplements were superior. We also performed subgroup analysis according to NOS score and for dementia we found a more significant decrease in studies with ≤ 7 points; for AD, the decrease was more significant in studies with more than 7 points. We also performed subgroup analyses according to study type, and we found that case-control studies significantly reduced the risk of dementia than cohort studies; cohort studies reduced the risk of AD more significantly. In conclusion, we found that either high dietary intake or vitamin E supplementation reduced the risk of dementia. We recommend that older adults should eat more foods rich in vitamin E or oral vitamin E supplements to prevent the development of dementia.

Vitamins are essential elements in maintaining the physiological functions of the brain. Its antioxidant function, which can reduce oxidative stress in dementia pathology (Tadokoro et al., 2020; Ali et al., 2021), can also react with free radicals, thereby preventing hippocampal neuronal apoptosis caused by reperfusion after ischemia and playing a role in protecting the brain (Tagami et al., 1999). Vitamin E has been found in animal studies to reduce the harmful effects of -amyloid and improve cognitive function in rodents (Rota et al., 2005; Montiel et al., 2006). Although the ideal timing of antioxidant effects is uncertain, research shows that antioxidants have an impact on dementia in its early stages (Berr et al., 2000; Nunomura et al., 2001; Praticò et al., 2002). Vitamin E's neuroprotective effect was confirmed in several experimental studies, which found that it prevented amyloid-induced cell injury and death in rat hippocampal cells (Behl et al., 1992), and slowed the progression of high-level amyloid-mediated neurological impairment in transgenic mice expressing human amyloid variants (Hsiao, 1995). Vitamin E intake increases the growth of natural killer cells in the elderly, And has been demonstrated in human dietary studies. Rotterdam's study of 5,395 people aged 55 years or older showed that participants with the highest dietary vitamin E content had a significantly reduced risk of dementia (De la Fuente et al., 2008).

We found no significant heterogeneity in the pooled results for vitamin E and dementia risk. Heterogeneity plays an essential role in meta-analysis. First, our study was less heterogeneous. Second, we further subgroup analyzed by dementia type, study type, NOS score, making our conclusions more specific. Third, according to the sensitivity analysis, our findings are stable and have some credibility. We must acknowledge that studies included in the meta-analysis have more or less some publication bias. For prospective cohort studies, the main sources of bias are selection bias, lost to follow-up bias, confounding bias. The main sources of bias regarding case-control studies are recall bias, choice bias, and confounding bias. We performed an offset test of the included studies using the Begg's funnel plot and Begg's test, and the results showed that no significant publication offset was found.

For the first time, we show a link between vitamin E and dementia risk. In a previous meta-analysis, Wang et al. (2021) investigated the connection of vitamin E supplementation with the risk and progression of AD and did not conclude that vitamin E supplementation reduces the risk of AD. It may be due to the small number of articles included in this meta-analysis and the low quality of the studies, which makes the results of the studies possibly biased. Our meta-analysis included a large number of literatures, with high study quality and reliable results. Moreover, we investigated high intake of vitamin E in diet, supplements with risk of dementia, not limited to supplements or AD, so our study is more persuasive. The meta-analysis conducted by li et al investigated dietary vitamin E with AD risk and concluded that dietary vitamin E intake contributes to the risk reduction of dementia (Li et al., 2012). This is consistent with the conclusions of our study. However, our study is of high quality, has a wide range of studies, and is more scientific. Therefore, we can reduce the prevalence of dementia by vitamin E supplementation.

The advantages of our study, first, we are the first to propose an effect of vitamin E on the risk of dementia. Secondly, we not only search the English database, but also search the references of reviews and meta-analyses, so that the retrieved literatures are more comprehensive. Third, the results of the prospective cohort study we included are reliable. Fourth, our study had a larger sample size and the findings were more representative. Finally, our study had no significant publication bias, and the heterogeneity was low and the results were stable.

Our research has its own limitations. About all, we included publications written in English, that could have resulted in selection bias. Next, our study was not subgroup analyzed by territory, ethnicity, and gender. Third, no specific vitamin E dose was provided and there was no dosages meta-analysis. Fourth, the dose conversion tool was inconsistent between meals and vitamin E that we included in the study. Fifth, even when the study results are adjusted for variables, other variable factors may also influence the reliability of the findings.

This paper shows that high doses of vitamin E have a role in reducing the risk of dementia. Therefore, people with a family history of dementia can be given high-dose vitamin E to prevent the occurrence of dementia. However, we should also pay attention to the side effects of vitamin E. Studies have shown that long-term daily administration of vitamin E more than 400–800 mg can cause headache, dizziness, blurred vision, elevated blood pressure, hormone metabolism disorders, etc., and significantly aggravate the symptoms of patients with diabetes and heart disease (Roberts, 1981; Satia-Abouta et al., 2003). Due to the absorption and transformation of vitamin E in the liver and metabolism in the kidney, long-term massive administration of vitamin E can increase the liver and kidney load, leading to liver and kidney damage (Miller et al., 2005; Ribeiro et al., 2018). In summary, we should control the dose of vitamin E within a certain range to reduce its toxic side effects.

## Conclusion

High intakes of diet or vitamin E supplements can significantly reduce the risk of dementia. Therefore, the elderly can reduce the risk of dementia by appropriately increasing foods rich in vitamin E, but also pay attention to the toxic side effects of vitamin E. Although our research results are reliable, they should be further validated by large RCTs.

## Data availability statement

The original contributions presented in the study are included in the article/Supplementary material, further inquiries can be directed to the corresponding author/s.

## Author contributions

RZ and XH conceived the study and wrote a draft. HZ and JL performed the literature search. MZ and WZ extracted the required data. SJ and RL performed the statistical analyses. HY and HC reviewed the paper. All authors contributed to the article and approved the submitted version.

## Funding

This work was supported by grants from the Task Letter of Science and Technology Plan Project of Gansu Province (21JR1RA020, 21JR11RA194, and 21JR7RA597).

## Conflict of interest

The authors declare that the research was conducted in the absence of any commercial or financial relationships that could be construed as a potential conflict of interest.

## Publisher's note

All claims expressed in this article are solely those of the authors and do not necessarily represent those of their affiliated organizations, or those of the publisher, the editors and the reviewers. Any product that may be evaluated in this article, or claim that may be made by its manufacturer, is not guaranteed or endorsed by the publisher.

## Supplementary material

Table S1 We uploaded the data sheets of included studies in the attachment section.
